# Developments in oral health care in the Netherlands between 1995 and 2018

**DOI:** 10.1186/s12903-020-01174-8

**Published:** 2020-07-08

**Authors:** Joost C. L. den Boer, Wil J. M. van der Sanden, Josef J. M. Bruers

**Affiliations:** 1 Department of Social Dentistry, Academic Centre for Dentistry Amsterdam (ACTA), University of Amsterdam and Vrije Universiteit Amsterdam, Amsterdam, The Netherlands; 2 Department of Research & Information, Royal Dutch Dental Association (KNMT), Utrecht, The Netherlands; 3 Department of Dentistry, Quality and Safety of Oral Healthcare, Radboud Institute for Health Sciences, Radboud University Medical Center, Nijmegen, The Netherlands

**Keywords:** Legislation and regulations, Competence, Professional development, Remuneration, Funding, Collaboration, Manpower, Work and practice situation, Amount and nature of care

## Abstract

**Background:**

Over the past several decades, changes in legislation and regulations have been implemented in oral health care in the Netherlands. In 1995, for example, a major transformation in the funding of oral health care was implemented, after which most oral health care for adults was no longer covered by national insurance. In 1997, the Individual Healthcare Professions Act, in which the authorizations of care providers were described, was established. The Healthcare Quality, Complaints and Disputes Act, established in 2016, concerns the accountability of professional behavior. Regulations concerning employment have changed several times since 1995. These changes have affected the work and practice situation of oral health care providers.

**Methods:**

Data from many publicly available sources were gathered and combined with internal reports mainly derived from the Data Stations project of the Royal Dutch Dental Association. This project was established in 1995 and, since its initiation, 6716 dentists have participated an average of 6.7 times.

**Results:**

Between 1995 and 2018, nearly all professional groups in oral health care increased, particularly those of dental hygienists and prevention assistants. The number of dental practices decreased, but practices got larger in terms of dental units, number of patients, and personnel. The percentage of inhabitants visiting oral health care professionals remained unchanged, but the type of care provided moved towards more prevention. Oral health care providers exploited new opportunities to enhance and express their professional behavior.

**Conclusions:**

Oral health care in the Netherlands has evolved in recent years toward more collaboration in teams, and professions have established institutions to promote the quality and safety of care. Greater emphasis has been placed on prevention of dental diseases. These processes were influenced by new legislation and regulations, demographic changes within professional groups, and other social developments.

## Background

In recent decades, changes in legislation and regulations have been implemented in various areas of health care in the Netherlands. These changes have affected the work situation of dentists and other oral health care providers related, in particular, to their competence, the funding of oral care, the collaboration between oral health care providers, and the accountability for professional behaviour.

### Competence

The authorizations of care providers in the Netherlands are formulated in the Individual Healthcare Professions Act (IHPA), which was established in 1997 [1]. Under this act, care providers are divided into different groups. Each group has its own requirements concerning qualifications, authorizations, and responsibilities. Five types of oral health care providers are covered by the IHPA: Oral- and maxillofacial surgeons (OMFSs), orthodontists, dentists, dental hygienists, and dental prostheticians. Dentist is a protected professional title; only persons registered in the IHPA-register are entitled to use this title. This also applies to dental specialists, OMFSs, and orthodontists, for which additional registration requirements apply. A specific group are differentiated dentists, who are not specialists but have specialised in a specific area of dentistry by means of extra training. A differentiation training is recognised by a scientific association, is usually part time, and lasts, on average, 3 years. Some differentiated dentists work exclusively as such, and others also work as general dentists. The Royal Dutch Dental Association (KNMT) recognises eleven differentiations: anxiety counselling dentistry, care for the disabled, endodontology, geriatric dentistry, gnathology, maxillofacial prosthesiology, oral implantology, pedodontology, periodontology, restorative dentistry, sleep medicine dentistry.

Dental hygienists and dental prostheticians are educational titles. For these professions, registration in the register for healthcare providers (IHPA-register) does not apply; however, the professional organisations of both professional groups established a register under private law. A five-year ‘task redistribution experiment in oral health care will’ start in 2020. In this experiment, dental hygienists are allowed to indicate and treat primary dental carious cavities, administer local anaesthesia, and indicate and take intraoral X-ray without the intervention of a dentist. To make this possible, a temporary extension for oral hygiene will be added to the IHPA-register.

The IHPA is not applicable to dental technicians and dental assistants, who do not have an authorization to provide oral health care independently. Similarly, the IHPA is not relevant to those trained as so-called prevention assistants by means of additional courses, which have been in existence since 1995. Prevention assistants are allowed to treat patients under conditions of task delegation. Table 1 provides an overview of the oral health care providers in the Netherlands, their training and their authorizations.

**Table 1:** Oral health care professions in the Netherlands

profession	training	authorised treatment (1)
dentist	dentistry (MSc)	reserved procedures: ^a^ - surgical procedures - assessment of injections - administration of anaesthesia - assessment of intraoral X-rays - prescription of medicines
orthodontist	dentistry (MSc) + specialisation dento-maxillary orthopaedics	reserved procedures: - surgical procedures - assessment of injections - administration of anaesthesia - assessment of intraoral X-rays - prescription of medicines
oral and maxillofacial surgeon	dentistry (MSc) + medicine (MSc) + specialisation oral and maxillofacial surgery	reserved procedures: - surgical procedures - assessment of injections - administration of anaesthesia - assessment of intraoral X-rays - prescription of medicines
dental hygienist	dental hygiene (BSc)	without referral or delegation: - dental cleaning practical autonomy: ^b^ - assessment of ionising radiation as part of examination - assessment of local anaesthesia by injection - treatment of primary cavities by preparation as part of restauration using mouldable materials
dental prosthetist	dental prosthetics (BSc)	without referral or delegation: - assessing dental prosthesis
prevention assistant	dental assistant (vocational education) + training prevention assistant	only delegated procedures ^c^
dental assistant	dental assistant (vocational education)	only delegated procedures
dental technician	dental technician (vocational education)	only delegated procedures

^a^ Reserved procedures are procedures that a professional is authorised to perform autonomously

^b^ Practical autonomy applies to procedures a professional can perform after referral according to protocol. Both referrer and performer must ascertain the competence of the performer. The patient must give consent for treatment by performer

^c^ When delegated procedures are performed, the delegating professional must always be available to intervene or assist when necessary. Both delegating professional and performer must ascertain the competence of the performer

In two advisory reports, published in 2000 and 2006, task redistribution in oral health care was encouraged [2, 3]. The Advisory Committee on Capacity in Oral Health Care (ACAO) released its advisory report in 2000, in which the focus was mainly on working in teams. According to ACAO, such teamwork would further enhance oral health care in the Netherlands [2]. Within a team, patients are treated by the oral health care provider whose knowledge and experience best match the care needed. The advisory report of the Committee on Innovation in Oral Health Care (CIO), released in 2006, provided advice similar to that of the ACAO but focused more on the further redistribution of tasks, primarily between dentists and dental hygienists [3]. The recommended changes included an expansion of the training curricula and, in the long term, a change in the dentist-dental hygienist ratio. Moreover, since 2006, dental hygienists have been directly accessible by patients for the provision of preventive oral health care, without referral by a dentist [4].

### Professional accountability

Since 2007, dentists have had the opportunity to voluntarily register these efforts in the Quality Register for Dentists (QRD) and to make these efforts visible to patients and others [5]. In 2008, the Quality Register for Dental Hygienists (QRDH) was established [5]. Both registers are subject to registration and re-registration requirements in terms of work activity and participation in knowledge- and skill-building activities (continuing professional development). The registration of activities related to knowledge and skills development will eventually become compulsory in the IHPA-register.

Another development in professional practice relates to the prevailing belief that health care providers should base their clinical practice, as much as possible, on the best available evidence. In 2012, the Health Council of the Netherlands recommended, among other things, the development of more evidence-based guidelines and encouraged widespread utilisation of these guidelines [6]. In response to this advice, in 2016, an Institute of Knowledge Translation in Oral Care, called ‘het KIMO’, was established. This is a national institute dedicated to the development and implementation of clinical practice guidelines to support oral health care providers in the provision of care according to best available evidence [7].

In 2016, important components of the legislation and regulations relating to accountability for professional behaviour were brought together in the Healthcare Quality, Complaints, and Disputes Act (HQCDA) [8–10]. HQCDA focuses primarily on actions taken when something goes wrong in the provision of care. For example, under HQCDA, care providers are required to be affiliated to a complaint procedure. However, professional accountability also implies making efforts to keep one’s knowledge and skills up to date.

### Remuneration

In general, there are three ways in which dentists are remunerated in the Netherlands; these are through practice ownership, through independent contracting, and through an employment contract. The laws and regulations concerning self-employment, including independent dentists without their own practice, have been changed a number of times in recent years. These changes were brought about due to the fact that the proportion and number of self-employed workers in the Dutch labour force increased, but, in many cases, the claim of self-employment was false [11]. In case of false self-employment, a freelance contract is actually an employed contract in disguise, because the self-employed is highly dependent on a client. However, because of the self-employment construction, the ‘self-employed’ cannot invoke employees’ rights. This apparent independence of self-employed workers has increased in at least eight European countries [12]. The main objective of the legislation and regulations was to simultaneously enable self-employment and prevent false self-employment [13]. Both independent contractors and dentist practice owners are affected by the changes in legislation and regulations.

### Funding

In 1995, the funding of oral health care for Dutch adults was radically changed [14]. Until then, people who earned less than a certain income were covered for medical and oral health care by a national health insurance. After 1995, the larger part of oral health care for adults was no longer covered by the national health insurance. All adult patients had to pay for most oral health care themselves, optionally by means of a voluntary, supplementary insurance. In 2006, this national insurance was replaced by the Health Insurance Act, which currently remains in effect. By this act, all residents of the Netherlands are obliged to take out standard health insurance for a limited range of medical care [15]. This range of care covered by basic insurance is determined by the government. For young residents, up to and including 17 years of age, the basic insurance covers the costs of preventive and curative (primary) oral health care. For adults, the reimbursement for oral health care is limited to prosthetic facilities in the edentulous jaw and dental care for people with severe physical and/or mental disabilities. It should be noted that, from 2008 through 2010, persons up to and including 21 years of age were considered young residents.

Furthermore, maximum rates applied to all oral health care through all years, with the exception of 2012, when an experiment with free price setting was conducted. Although the experiment was originally planned to last 3 years, after 6 months, a decision was made to terminate the experiment after 1 year.

The 1995 change in the funding of oral health care caused major changes for dentists, especially due to the fact that reimbursement for oral health care from the national insurance plan was associated with a mandatory half-yearly routine oral examination. This obligation applied to the vast majority of the adult population. The change in the funding prompted the professional organisation of dentists, KNMT, to initiate a continuous research project to monitor the consequences of this change [16, 17]. The studies in this so-called Data Stations Project (DSP) focus on the amount and nature of oral health care provided, on the work and practice situation of dentists, and on the views of dentists on their professional activity. In 2020, DSP is still active. Data from the project supports KNMT to represent its members and to substantiate its policy. KNMT is not unique in its research activities. In other countries, professional dental associations also encourage initiatives to promote structural research [18–20]. The nature of these research projects varies between countries.

In more than twenty years, via the DSP a multitude of data was collected regarding the changes described above and other subjects. These changes have greatly affect the professional practice of dentists, dental specialists, dental hygienists, and other oral health care providers. In this manuscript, we describe changes in professional practise in several professional groups in oral health care in the Netherlands. Specifically, we explore developments that occurred between 1995 and 2018 regarding: (a) manpower in oral health care, (b) dentists’ work and practice situation, and (c) the amount and nature of the care provided in general dental practices.

## Methods

Much of the data were collected within the aforementioned DSP [16, 17]. Data also come from the KNMT dentists’ database, registers of various scientific associations, and studies by the Dutch government.

### Data stations project

Within the DSP, research is carried out on dentists in the Netherlands, both KNMT members and non-members. The project consists of four sub-studies, which focus on ‘care provided’, ‘work situation and practice organisation’, ‘views on professional practice’, and ‘recently graduated dentists’. All examinations are carried out periodically in samples of dentists that are similarly extracted. Most samples consist (partly) of a panel of dentists who have registered for participation or who have participated in the previous edition of the study. Usually, an additional sample of dentist is added in order to guarantee the size and representativeness of the research sample. Data from three sub-studies were used. These sub-studies are described below.

- For the Dental Consumption Study (DCS), data are collected on the treatments that dentists have performed on a random 25% sample of their patients. These data have been gathered since 1997, systematic annual data are available from 1998 onwards. This data collection is facilitated by specific modules in the most commonly used software for dental practices in the Netherlands. These modules allow dentists to provide anonymised data on patient characteristics (gender, date of birth an insurance) and the universal codes of the dental treatments performed and claimed.
- The Dental Practice Survey (DPS) is a biannual survey about the work situation and practice organisation of dentists. The survey was first carried out in 1995. Through a questionnaire, dentists are questioned about, among other topics, the number of working hours, the composition of the dental team and patient population, the equipment available in practice, and the workload and waiting times for different types of treatment. Initially, only written questionnaires were used, which were sent out and gathered by post. Since 2011, dentists have also had the opportunity to participate via web survey. However, to date, written responses still exceed the internet response. Therefore, questionnaires are still sent out and gathered by post. Both written and web surveys include a respondent number, which can be used to check for double participation.
- The Survey of Recently Graduated Dentists (SRGD) was established in 2007 and is, in principle, conducted every 3 years. In SRGD, recently graduated dentists are asked about their first work experiences as dentists and their plans for future professional practice. Invitations for the first edition of this web-based questionnaire were sent by email to all dentists who had graduated in dentistry from a Dutch university in the 3 years prior to the survey. In the following editions, the ‘newly’ graduated of these institutions were invited. Due to the length of the questionnaires, the 2018 edition of SRGD was carried out in two phases. The first phase focused on the dentists’ assessment of their training; the second part focused on their work situation and their wishes for their future professional practice.

Between 1995 and January 2019, 6716 dentists participated at least once in DSP research. On average, individual dentists participated 6.7 times; thus, the total number of participations surpasses 45,000. Table 2 provides an overview of the number of editions of and the number of participants in the four sub-studies of the DSP. The results of the individual editions of all DSP studies are reported in internal research reports, in scientific and professional journals and on a website about the Dutch oral health care: www.staatvandemondzorg.nl. In this study, no data were used from the fourth sub-study, the so-called Omnibus Survey. This sub-study focusses on ‘views on professional practice’.

**Table 2:** Overview of data collection in the studies carried out within the Data Stations Project between 1995 and January 2019

	*start date*	*cross- sectio-nal (CS) and/or longitu-dinal (L)*	*number of editions*	*number of dentists who participated at least once*	*mean number of participations*^b^	*mean response percentage*^c^	*mean number of respondents*^d^
Dental Consumption Survey (DCS)	^a^ 1998	CS + L	21	1.513	9,1 (6,3)	80,9%	653
Dental Practice Survey (DPS)	1995	CS + L	29	3.500	3,8 (4,7)	54,1%	454
Omnibus Survey	1995	CS	40	3.858	4,4 (5,3)	56,6%	427
Survey of Recently Graduated Dentists (SRGD)	2007	CS + L	4	998	1,1 (0,4)	34,1%	286
Total	1995	CS + L	94	6.716	6,7 (8,5)	^e^ 60,3%	^e^ 480

^a^ First year of digital data collection

^b^ Standard deviation in parentheses

^c^ Calculated on the basis of the mean of the response percentages of all individual editions

^d^ Calculated on the basis of the mean number of respondents of all individual editions

^e^ Calculated on the basis of the figures in this table

### KNMT dentists’ database

Data on the size and composition of the professional groups of dentists, OMFSs, and orthodontists originate from the KNMT dentists’ database, in which information is recorded of all dentists and dental specialists authorised in the Netherlands. This database is filled with data regarding the graduates of all Dutch dentistry trainings, the Register of Dental Specialists, and applications for KNMT membership. For verification purposes, the data are compared with the IHPA-register. Registration in this register is mandatory to be authorised to work as a dentist, OMFS, or orthodontist in the Netherlands. The KNMT database contains background characteristics, including sex, date of birth, date and place of graduation, place of establishment, and registration in QRD. In addition, several types of data related to KNMT membership are kept, including activity within the oral health care sector. However, these data are only reliable for KNMT members. Therefore, the dentists’ database does not automatically provide an accurate picture of the sizes of the active professional groups. Therefore, the number of active dentists and dental specialists is defined as a specific part of all dentists and dental specialists in the dentists’ database, namely all dentists and dental specialists aged 64 years and younger of whom KNMT knows a home address and/or a work address in the Netherlands.

### Other information on oral health care providers

The data on differentiated dentists are derived from the scientific associations that recognize and register the differentiations in question. These data are not always reported publicly by the associations themselves; in some cases the data were made available to and published by third parties [5, 21–36]. The number of dental practices is a rough estimate based on the number of active dentists and the number of dentists per practice. The number of dental practices affiliated with a dental chain was estimated based on data from www.tandarts.nl. According to the owners, this website offers an overview of all dental practices in the Netherlands. In two news items on this website, the total number of dental practices listed is stated as well as the percentage of these practices that are affiliated with a dental chain [37, 38].

### CBS data

Data on the number of inhabitants of the Netherlands, their oral health care status, and their visits to oral health care providers were derived from the Central Bureau of Statistics (CBS) [39]. As are data on total expenditures oral health care and gross domestic product (GDP) [39]. CBS is the government body responsible for the collection and maintenance of statistical data. CBS has access to data from the digitalised population records of all municipalities and therefore can provide information about the number of inhabitants of the country. Based on this number and on data from the KNMT dentists’ database, the number of inhabitants per dentist was calculated.

Data on the number of visits to dentists, orthodontists, and dental hygienists were retrieved from the CBS Health Survey, an annual survey among a sample of 15,000 inhabitants of the Netherlands. From 1997 to 2009, this survey was part of the Permanent Survey of Living Conditions.

## Results

### Professional groups in oral health care

Table 3 shows that the number of dentists and dental specialists in the Netherlands aged 64 years and younger increased between 1995 and 2018, as did the number of dental hygienists and dental prostheticians. The number of dental technicians decreased during this period. The combined number of dental and prevention assistants increased in the aforementioned period, and, within this group, the proportion of prevention assistants grew. In many cases, prevention assistants work in a combined form, as they also perform tasks as a dental assistant.

**Table 3:** Developments in professional groups in oral health care in the Netherlands between 1995 and 2018

	1996	2001	2006	2011	2016	2018
number of dentists	6.887	7.397	7.994	8.590	8.656	8.794
proportion of female dentists	18%	21%	26%	32%	40%	43%
proportion of dentists aged 39 years or younger	nda	28%	26%	31%	35%	37%
proportion of dentists 40–49 years	nda	38%	32%	21%	19%	20%
proportion of dentists 50–59 years	nda	28%	34%	34%	30%	27%
proportion of dentists 60 years or older	nda	6%	8%	14%	16%	16%
proportion of dentists who graduated abroad ^a^	nda	5%	7%	10%	14%	16%
**dental specialists**						
orthodontists	255	287	283	317	318	326
oral and maxillofacial surgeons	194	198	214	245	273	289
**differentiated dentists**^b^						
periodontologists	44	61	80 ^b^	84	81	87
dentists for maxillofacia prosthetics	nda	nda	nda	39	33	46
gnathologists	nda	45	47 ^b^	52	42	54
endodontologists	nda	26	37 ^b^	64	87	92
oral implantologist	–	–	188 ^b^	448	345	307
dentists for disabled patients	–	–	nda	35	22	30
geriatric dentists	–	–	15	18	17	19
pedodontologists	–	14	nda	41	51	56
anxiety counselling dentists	–	–	1	27	16	16
sleep medicine dentists	–	–	–	nda	116	125 ^b^
restorative dentists	–	–	–	–	35 ^#2^	36 ^b^
**other oral health care providers**						
dental hygienists	1.489 ^b^	1.789 ^b^	2.267	2.425 ^b^	3.216 ^b^	3.500 ^b^
dental prosthodonitists	nda	nda	293 ^b^	350 ^b^	330 ^b^	600 ^b^
dental technicians	nda	nda	nda	5.000 ^b^	4.700 ^b^	4.700^b^
prevention assistants	nda	nda	2.375 ^b^	6.300 ^b^	7.650 ^b^	8.000 ^b^
dental assistants ^c^	8.533 ^b^	nda	14.125 ^b^	11.900 ^b^	11.700 ^b^	11.200 ^b^
number of inhabitants (× 1.000.000)	15,5	16,0	16,3	16,7	17,0	17,2
number of inhabitants per dentist^a^	2.250	2.161	2.043	1.939	1.962	1.954

^a^ Including dentists for whom the place of graduation is not registered by KNMT; in practice, this nearly always applies to dentists who graduated outside the Netherlands

^b^ The data refer to the previous year

^c^ Excluding prevention assistants

- Differentiation was not yet recognised by KNMT

*nda* No data available

In 2000, four dental differentiations were recognised by KNMT: periodontology, maxillofacial prosthesiology, gnathology, and endodontology. Since then, seven more differentiations have been recognised: oral implantology, care for the disabled, geriatric dentistry, pedodontology, anxiety counselling dentistry, sleep medicine dentistry, and restorative dentistry. This extension of differentiations has led to an increase in the number of differentiated dentists.

Furthermore, the composition of the professional group of dentists has changed. The proportion of female dentists has increased since 1995. The age distribution has also changed over the years. Between 2001 and 2016, the proportion of dentists aged 60 or older increased from 6 to 16%, while the proportion of dentists aged 39 years or younger also increased between 2006 and 2018: from 26 to 37%. Proportionally, the middle age groups have become smaller. It is also striking that the proportion of dentists who graduated outside the Netherlands increased from 5 to 16% between 2001 and 2018.

### Practice organisation and collaboration

Over the years, dental practices have grown in size, and dental teams have expanded. Table 4, for example, shows that the average number of treatment units per practice has increased. The number of patients attending dental practices has increased accordingly, as well as the number of care providers and other employees. Whereas the size of practices increased, the number of practices decreased. Furthermore, in recent years, more practices have affiliated with a so called dental chain. The enthusiasm of recently graduated dentists to start their own practice seems to have declined. In 2007, 69% of then recently graduated dentists expressed a wish to eventually become a practice owner; in 2018, 54% did.

**Table 4:** Development in dental practices and collaboration in oral health care in the Netherlands between 1995 and 2018

	1996	2001	2006	2011	2016	2018
number of dental practices	5.700	5.700	5.800	5.600 ^a^	5.100 ^a^	4.600
number of dental units per dental practice	1,3 ^a^ (1,3-1,4)	2,0 (1,9-2,2)	2,3 (2,2-2,5)	2,5 (2,3-2,7)	3,1 (2,7-3,5)	2,8 (2,6-3,0)
number of ‘regular’ patients per dental practice ^b^	2.283 ^a^ (2.185–2.380)	2.703 (2.518–2.888)	2.899 (2.689–3.109)	2.916 (2.710–3.122)	3.298 (2.783–3.812)	3.000 (2.722–3.278)
number of patients visiting per week per dental practice	130 ^a^ (123–136)	141 (130–151)	151 (140–163)	149 (139–159)	169 (141–197)	157 (139–174)
number of dental practices affiliated with a dental chain	nda	nda	nda	nda	240	325
proportion of recently graduated dentists who want to become practice owner	nda	nda	69% ^c^	51% ^a^	nda	54%
**type of practice (based on collaboration with other dentists)**						
1 dentist practice owner, 0 dentist non-owner	nda	71%	63%	59%	52%	44%
1 dentist practice owner, 1+ dentist non-owner	nda	14%	18%	25%	25%	23%
2+ dentist practice owner, 0 dentist non-owner	nda	10%	14%	9%	13%	21%
2+ dentist practice owner, 1+ dentist non-owner	nda	5%	5%	7%	10%	12%
**proportion of dental practices employing one or more:**						
dental assistants ^d^	96%	94%	89%	89%	88%	90%
prevention assistants^d^	nda	19%	45%	47%	54%	54%
dental hygienists	28%	32%	35%	37%	46%	52%
secretary/administrative assistants	31%	39%	34%	46%	51%	43%
practice managers	nda	7%	19%	21%	28%	25%
dental technicians / dental prostheticians	nda	3%	5%	6%	6%	11%
**proportion of dentists in total practice formation**						
small (<= 25%)	nda	nda	14%	12%	12%	9%
rather small (26–40%)	nda	nda	41%	41%	45%	50%
rather large (41–55%)	nda	nda	34%	36%	33%	30%
large (> 55%)	nda	nda	11%	11%	10%	11%
**referral to dental hygienist(s)**^e^				a		
yes, namely to dental hygienist:^f^	nda	86%	89%	90%	91%	90%
- within own practice	nda	nda	*33%*	*36%*	*49%*	*55%*
- in dental practice of colleague	nda	nda	*11%*	*11%*	*9%*	*13%*
- in independent dental hygienist practice	*41%*	nda	*57%*	*60%*	*53%*	*47%*
no	nda	14%	11%	10%	9%	10%
**delegation to prevention assistant(s)**				a		
yes	nda	59%	48%	47%	52%	55%
no	nda	41%	52%	53%	48%	45%

^a^ The data refer to the previous year

^b^ Number of patients who visit the practice at least once a year

^c^ The data refer to the succeeding year

^d^ Until and including 1999, no distinction was made in DPS between dental assistants and prevention assistants

^e^ In 1995, only referral to an independent dental hygienist practice was surveyed. In 2001, the work situation of the dental hygienist to whom dentists referred was not surveyed

^f^ Multiple answers possible, the columns may add up to more than 100%

() Where applicable, 95%-confidence intervals are presented in between brackets

*nda* No data available

### Care provided and funding

Table 5 shows that, between 1996 and 2018, the number of inhabitants of the Netherlands increased from 15.5 million to 17.2 million. As the proportion of people with natural dentition also increased during this period, the total number of people with natural dentition increased more than the number of inhabitants. Moreover, the proportion of inhabitants contacting a dentist was rather stable between 2001 and 2018, fluctuating between 75 and 80% per year. In addition, in 2016 and 2018, about one third of the inhabitants of the Netherlands aged 12 years and older visited a dental hygienist, and 8% of those aged 8 years or older visited an orthodontist.

**Table 5:** Developments in oral health care provided, expenditure on oral health care, and activities to promote knowledge and skills of oral health care providers in the Netherlands between 1995 and 2018

	1996	2001	2006	2011	2016	2018
proportion of the population (1 year and older) visiting a dentist in the 12 months prior	74%	78%	78%	78%	79%	80%
proportion of the population (12 years and older) visiting a dental hygienist in the 12 months prior	nda	nda	nda	nda	32%	36%
proportion of the population (8 years and older) visiting an orthodontist in the 12 months prior	nda	nda	nda	nda	7%	8%
proportion of inhabitants with own dentitions	81%	84%	87%	nda	nda	nda
number of visits to dentists per patient per year	2,7	2,9	2,8	3,0	3,2	3,4
number of visits to dentists per inhabitant per year ^a^	2,0	2,3	2,2	2,3	2,6	2,8
**type of treatment**	**b**					
consultation and diagnostics	33%	30%	20%	18%	17%	15%
consultation and diagnostics and prevention	15%	19%	27%	33%	36%	37%
consultation and diagnostics and curation	33%	30%	20%	16%	15%	14%
consultation and diagnostics and prevention and curation	15%	18%	29%	29%	28%	29%
prevention and/or curation (emergency care or treatment of pain complaints)	4%	3%	4%	4%	4%	5%
expenditure on oral health care in dental practices (× 1 million)	nda	1.519	2.025	2.682	2.877	2.897
expenditure on oral health care in dental practices, as a percentage of GDP	nda	0,32%	0,35%	0,42%	0,39%	nda
proportion of dentists registered in QRD	–	–	–	40%	51%	51%
**number of hours per month spent by dentists on activities to promote knowledge and skills:**						
continuing education	nda	5,0 (4,6-5,4)	4,8 (4,3-5,3)	6,3 (5,8-6,8)	5,1 (4,4-5,8)	4,5 (4,1-5,0)
peer learning exchanges	nda	1,5 (1,3-1,7)	1,7 (1,4-2,0)	2,1 (1,9-2,4)	1,8 (1,5-2,1)	1,4 (1,1-1,6)
reading professional literature	nda	5,3 (4,8-5,8)	4,7 (4,2-5,1)	5,0 (4,7-5,4)	4,5 (4,1-5,0)	4,2 (3,6-4,7)

^a^ Including all inhabitants who have not visited the dental practice

^b^ The data refer to 1998

() Where applicable, 95%-confidence intervals are presented in between brackets

nda No data available

The total costs for oral health care increased by about 1.4 billion Euro between 1996 and 2018, to a total of almost 2.9 billion Euro. The relative costs for oral health care also increased, especially between 2001 and 2016.

In recent decades, there has been a change in the type of oral health care provided. In 1998, about one third of all patients were charged for prevention; by 2018, about two thirds of all regular patients were charged for prevention In this period, the proportion of patients charged for curation, declined from 48 to 43%. In this comparison the care to non-regular patients, who visited the practice for emergency care and treatment of pain complaints, was excluded.

### Professional accountability

In 2011, 4 years after its foundation, 40% of dentists were registered in the QRD. The proportion of dentists registered in the QRD increased to 51% in 2016 and remained the same after that year. No data are available on registrations in the QRDH. The number of hours dentists spend per month on quality-enhancing activities remained fairly stable between 2001 and 2011.

## Discussion

The results described above show that the professional practice of oral health care providers has changed in several respects in recent decades. These changes can be linked to social developments within the health care sector in the areas of competence, professional development, remuneration, and funding.

### Manpower, work, and practice situation

The IHPA offers various opportunities for referral and task delegation. Within oral health care, response to these opportunities by care provider has results in a substantial increase in collaboration in the provision of oral health care in the Netherlands. Oral health care is no longer provided mainly by general dentists; instead, care is also provided by differentiated dentists, dental hygienists, prevention assistants, and dental prostheticians. Two advisory committees saw the expansion of collaboration as an opportunity to improve the quality of care and solve the shortage of oral health care providers [2, 3]. According to these committees, referral and task delegation would ultimately lead to a reduction in the required number of dentists and an increase in the number of oral hygienists. So far, things have been different. Almost all professional groups in oral health care have grown, and the increase in the number of dental hygienists as well as, in particular, the emergence of prevention assistants stand out. Especially for prevention assistants, opportunities were created by task shifting. Jerković-Ćosić et al. found that dental hygienists with four-year training are given a more extensive range of tasks than their colleagues with a two- or three-year training [40]. The increased focus on complex tasks by dental hygienists allows the prevention assistant to carry out the more basic primary and secondary prevention tasks. It is unclear to what extent the current training of dental assistants responds to these possibilities to broaden the range of tasks.

Other factors also provided an incentive for more collaborations. Due to the sharp increase in female students and regionally experienced shortages of dentists, the composition of the dental profession changed. The proportion of female dentists increased significantly, as did the proportion of dentists who graduated outside the Netherlands. In addition, the number of dentists specialising in a specific area of dentistry has increased. Partly because of this changing composition of the profession, the number of dentists active in their own practice decreased. Female dentists and dentists who graduated abroad appear to be less inclined to start their own practices [41–43]. The increased collaboration in larger dental teams has led to the development of larger practices and more affiliation with dental chains, among other things. The fact that, in the last decade, a relatively large number of older dentists have ended their practice, which were, in some cases, difficult to sell, has also contributed to this shift. Moreover, these changes have not yet led to a solution to the problem of dentist shortages. Particularly in certain regions, dentist shortages were reported in 2019 [44].

### Care provided

In the changing landscape of dental practices, the proportion of inhabitants visiting a dentist and/or dental hygienist remained unabatedly high. In fact, this proportion is so high that there is not much room for increase as a proportion of the people do not visit a dentist for various reasons [45]. However, there was a shift to more preventive care. This may be triggered by the improved oral health of, especially, Dutch adults, which reduced the need for curative treatment [46]. Attention to quality and safety of care also increased [47]. Evidence-based clinical practice has been better facilitated by the establishment of an independent institute for the development of clinical guidelines within oral healthcare by the oral health care professions [7, 48]. Furthermore, dentists and dental hygienists have increased opportunities to display their efforts to keep their knowledge and skills up to date. A significant proportion of professionals seizes these opportunities. For dentists, this does not seem to elevate (or alleviate) the time they spend on continuing education, participation in study groups, or reading professional literature.

### Restrictions

A large portion of the data used for this study was obtained for DSP and, therefore, primarily concerns dentists. The fact that less information is presented in this study about other professional groups is due to limited availability of data. Moreover, most of the care provided by dental hygienists and prevention assistants is performed in dental practices and therefore included in DCS. For this reason, the consequences of this omission are limited [49].

The developments described only concern the Netherlands and therefore cannot be compared automatically with developments in other countries. After all, legal frameworks differ between countries, as do the ways in which these framework affect the provision of care [50, 51]. However, the description of specific developments in the Netherlands in recent years may provide insights for other countries. In the Netherlands, for example, the possibilities for task delegation and referral are strongly embedded in legislation and regulations. This study provides a scenario for the consequences of this policy choice for countries where the authorisation of different professional groups is debated. Additionally, there are, of course, also similarities between countries, as certain developments and challenges are cross-border in nature. The focus on evidence-based clinical guidelines and the establishment of an institution to develop them, for example, can be observed in several other countries as can a similar emergence of dental chains [52–56]. Furthermore, in many countries teamwork in oral health care practices is increasing and task redistributions is under consideration or construction [57–59]. In short, this study provides a useful outline of these cross-border developments from a Dutch perspective.

## Conclusion

Influenced by new legislation and regulations, demographic changes within professional groups, and other social developments, oral health care in the Netherlands has evolved in recent years toward more collaboration in teams, and professions have established institutions to promote the quality and safety of care. In addition, emphasis in the provision of oral health care has been placed even more on prevention of oral diseases.

## Data Availability

The internal research reports are available from the corresponding author upon reasonable request.

## Abbreviations

ACAO: Advisory Committee Capacity Oral Health Care *(In Dutch: Adviescommissie Capaciteit mondzorg)*
CBS: Central Bureau of Statistics *(Centraal Bureau voor de Statistiek)*
CIO: Committee on Innovation in Oral Health Care *(Commissie Innovatie Mondzorg)*
DSP: Datastations Project *(Project Peilstations)*
GDP: Gross Domestic Product
KNMT: Royal Dutch Dental Association *(Koninklijke Nederlandse Maatschappij tot bevordering der Tandheelkunde)*
QRDH: Quality Register for Dental Hygienists *(Kwaliteitsregister Mondhygiënisten)*
QRD: Quality Register for Dentists *(Kwaliteitsregister Tandartsen)*
SRGD: Survey of Recently Graduated Dentists *(Onderzoek Recent afgestudeerde Tandartsen)*
OMFS: Oral- and maxillofacial surgeon
DCS: Dental Consumption Survey *(Onderzoek Tandheelkudndige Consumptie)*
DPS: Dental Practice Survey *(Onderzoek Tandheelkundige Praktijkvoering)*
IHPA: Individual Healthcare Professions Act *(Wet op de beroepen in de individuele gezondheidszorg)*
HQCDA: Healthcare Quality, Complaints, and Disputes Act *(Wet kwaliteit klachten en geschillen zorg)*
